# Microbial associations and transfers across the One Health Triad effects on human and animal adiposity and temperament: a protocol for an observational pilot study

**DOI:** 10.3389/fpubh.2023.1225188

**Published:** 2023-09-07

**Authors:** Mariah K. Zeigler, Kiley B. Vander Wyst

**Affiliations:** ^1^Master of Public Health Program, College of Graduate Studies, Midwestern University, Glendale, AZ, United States; ^2^Clinical Research Support Team, Anschutz Medical Campus, University of Colorado Denver, Denver, CO, United States

**Keywords:** One Health, microbiome, complementary feeding, adiposity, temperament

## Abstract

**Introduction:**

It is known that humans and pet dogs harbor microbial communities that are important regulators of health and disease. Pet dogs have been shown to promote microbial exchange between members of a household, a process that may have lasting health implications. Infancy marks a unique period of development as environmental exploration and introduction to complementary foods occur. This may lead to greater opportunities for microbial transfer between pet dogs and human infants due to a more confined shared environment, similar means of mobility, greater physical contact, and increased frequency of shared foods. This human-animal bond has led to extensive research in the areas of childhood allergies and behavioral health; however, there is a paucity in the available literature that has evaluated how this unique ecological relationship may impact both human and animal health.

**Methods:**

Infants who reside in a household with a pet dog will be recruited from the greater Phoenix metropolitan area for this longitudinal, observational pilot study and followed through the complementary feeding period. Infant and pet dog fecal, salivary, and skin samples, as well as environmental samples from feeding areas/surfaces and main indoor play areas from both infants and pet dogs will be collected through in-home visits before (~5 mos), during (~9 mos), and after (~12 mos) the complementary feeding (CF) period. Anthropometrics, temperament, and dietary habits of both infants and pet dogs along with assessment of the home condition will also be collected. Microbial comparisons between infant and pet dog samples and evaluation of microbial changes during the CF period will be evaluated. Further, we will assess relationships between microbial composition and adiposity and temperament of both infants and pet dogs.

**Discussion:**

The proposed observational pilot study will advance the available science by exploring how microbial communities are associated and change between infants and pet dogs before, during, and after the CF period, a unique period of human growth and development. Findings from this study will provide insights into the impact these ecological relationships have on each other and how transfer across the One Health Triad impacts human and animal health.

## Introduction

Although not a new concept, One Health recognizes that the health of people is intimately tied to the health of animals and the environment (1). As humans evolved from nomadic hunter-gatherers to settled agriculturalists, their relationship with animals evolved from being principally for physical labor and food production to also include companionship (2). Domestication of animals as pets has changed the shared environment between humans and companion animals, including dogs, leading to more frequent and intimate interactions within a shared living environment (3). This unique relationship has led to significant benefits to humans’ emotional, physical, and social well-being (4, 5). There has been extensive research evaluating the positive impact the human-animal bond between humans and pet dogs has on human health, with research investigating the protective effect that pet dogs provide against allergies (6) and behavioral problems among pet owners dominating the literature (7, 8). However, this beneficial relationship may extend to other diseases across the One Health Triad, including scantly researched benefits to the companion animal.

It is well known that both humans and pet dogs harbor distinct microbial communities that serve a multitude of roles and are important regulators of health and disease (9–11). Recently, efforts have been extended to understand microbial similarities between pets and their owners (12), among humans (13) and pets (14) with their environment, and among members of the same household (15, 16). Pet dogs promote microbial exchange between members of the same household (15, 16). This may, in part, be due to more confined living environments having a greater effect on inhabitants’ microbiome (13, 14). Cohabitation affects microbial composition of both pets and humans across a variety of body epithelia with microbial exchange during specific periods of growth and development leading to greater health implications (13, 14).

Infancy marks an important period of growth and development as introduction to solid foods is associated with shifts in the dominant bacterial phyla present in the infant gut (17, 18), resulting in increased microbial diversity and a more stable GM (19). Infants have a unique relationship with their pets due to a more confined shared environment (20), similar means of mobility, and greater opportunities for microbial transfer through physical contact (21) and shared foods (1, 12, 22). Research has shown that pet ownership enriched the abundance of *Ruminococcus* among infants, a bacterial genus negatively associated with atopy (22). This immune benefit to human health due to pet exposure has been well studied, particularly related to allergies (15). However, whether there are similar health benefits of microbiome transfer to the animal or for other inflammatory diseases has not been extensively researched.

Perturbations in microbial composition of various body sites are associated with a wide range of human health issues including adiposity (23, 24), behavioral problems (8), and temperament (11). Similarly, microbial dysbiosis among pet dogs is associated with aggressive and phobic behaviors (25, 26) and overweight status (20). Therefore, it is important that we examine the impact ecological relationships have on microbial colonization and how transfer across the One Health Triad impacts human and animal health. This manuscript describes an observational pilot study designed to evaluate microbial composition and diversity associations between human infants, pet dogs, and the home environment, and their impact on adiposity and temperament in both the infant and pet dog before, during, and after the complementary feeding period.

## Methods/design

### Aims and design

**Figure 1:**
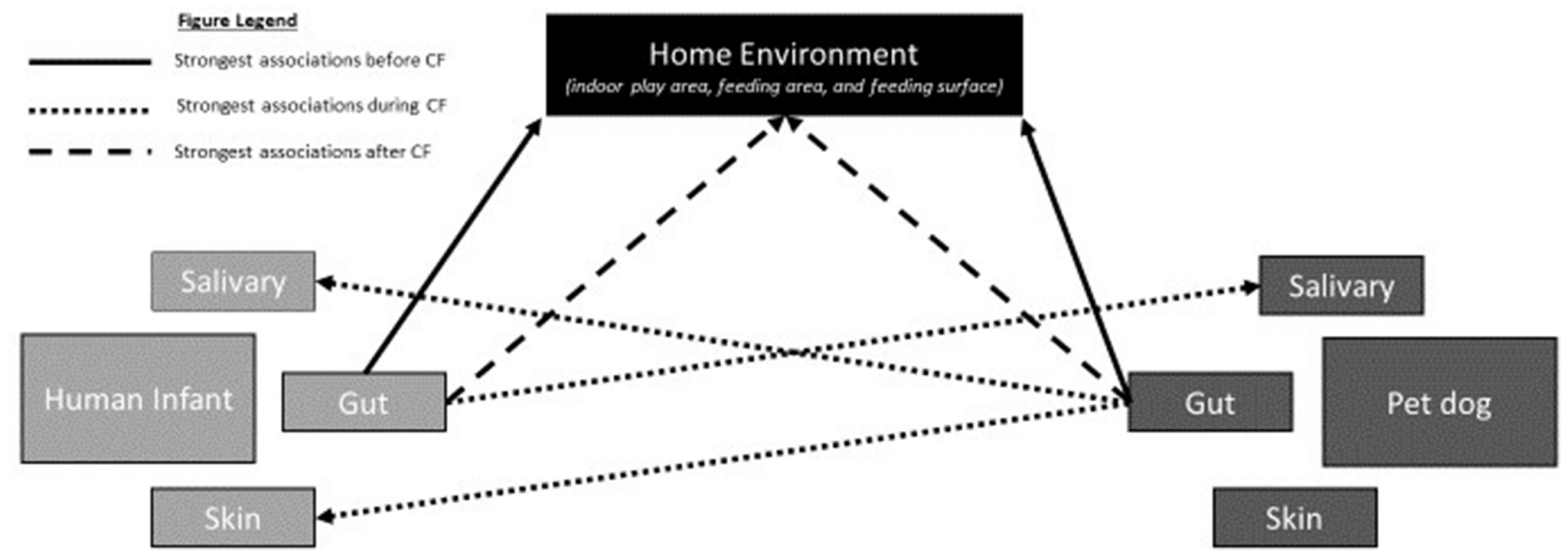
Graphical illustration of the proposed relationships in Aim 1 at each of the three timepoints. Before the complementary feeding (CF) period (solid black line), the human infants and pet dogs will have gut microbiomes (GM) that have the strongest association with their home environment. During the CF period (dotted black line), the human infant GM will have the strongest association with the microbial composition and diversity of the salivary microbiome of the pet dog and the pet dog GM will have the strongest association with both the infant salivary and skin microbiota during. After the CF period, the human infants and pet dogs will have a GM that will have the strongest associations with the microbial composition and diversity of their home environment.

This pilot study is designed as a 1-year longitudinal, observational study to assess microbial associations and transfer between human infants, pet dogs, and the home environment and the impact on human and animal adiposity and temperament. In this study, we aim to:

1. Investigate associations between the gut microbiome (GM) of human infants and pet dogs with the microbial diversity of the saliva, skin, and home environment before, during, and after the human complementary feeding (CF) period. We hypothesize that human infants and pet dogs will have gut microbiomes (GM) similar to their home environment before the CF period; the human infant GM will have the strongest association with the microbial composition and diversity of the salivary microbiome of the pet dog and the pet dog GM will have the strongest association with the infant salivary and skin microbiota during the CF period; and after the CF period the human infant and pet dog GMs will have the strongest association with the microbial composition and diversity of their home environment. See Figure 1 for graphical illustration of proposed relationships.
2. Evaluate the change in the microbial diversity of the GM of the human infant and pet dog before and during CF; and before and after CF. We hypothesize that there will be a significant change of gut microbiota in both the human infant and pet dog at both timepoints, and that these changes will be correlated with each other.
3. Assess whether the changes observed in Aim 2 are correlated with subsequent changes in adiposity and/or temperament. We hypothesize that the change in microbial composition and diversity of the gut in both the human infant and pet dog will be associated with subsequent changes in adiposity and temperament of both the human infant and pet dog, respectively.

### Participants and procedures

#### Subject eligibility

Inclusion criteria consists of a household with an infant between 4 and 6 months of age that has not received any complementary foods, has one pet dog that is over the age of 6 months that has been spayed or neutered, with a breed analysis over 50% for one or more of the following breeds (Labrador Retriever, Golden Retriever, German Shepard, and Poodle) (27), and consists of at least two cohabiting adults (e.g., partners and couples). Exclusion criteria includes households with an infant that has already received complementary foods, has been exposed to antibiotics in the last month either directly (28) or indirectly via breastmilk consumption (29), that were small- (30) or large-for-gestational age (31) at birth, born at <37 weeks gestation, have growth, chromosomal, or genetic abnormalities (32), have developmental delays, or severe comorbidities that could impact growth and development. Households that have more than one pet dog; or a pet dog that received antibiotics in the last month (33), have known history of being factious, do not have proof of rabies vaccination (34), have been diagnosed with predetermined comorbidities (35, 36), or have not been examined by a veterinarian at least once in the past 12 months will also be excluded. Additionally, households where any member smokes inside the home will be excluded (37). Households that meet the criteria for both infants and pet dogs will be included in the pilot study. If either the infant or the pet dog do not meet inclusion criteria the household will be excluded.

#### Study procedures

Potential eligible participants will be recruited from email and pilot study promotional materials distributed to Midwestern University faculty, staff, and students residing in the greater Phoenix metropolitan area. Individuals that are interested will be asked to contact the research team to assess eligibility. Caretakers will be screened over the phone to determine study eligibility. If deemed eligible and willing to participate, caretakers will be asked to provide a proof of current rabies vaccination and lack of aggressive or fractious behavior of the animal toward their veterinarian or unknown people in the home. Research staff will schedule the first in-home study visit only after receipt of proof of vaccination, predominant dog breed verification, and review of behavioral status of the animal. Informed consent will take place at the first in-home study visit. If the caretaker decides they do not want to participate during this visit, the visit will end, and research staff will have no future contact with the family. Caretakers will consent to at least three additional in-home visits by research staff. The first study visit will occur when the infant is between 4 and 6 months of age, before receipt of any complementary foods, the second at ~9 months of age, and the last when the infant is approximately 12 months of age and consuming the majority of their nutrients from semi-solid and solid foods.

At all home visits, research staff will assess demographic, health history, anthropometrics, dietary history, and temperament for both the infant and pet dog to ensure continued eligibility for inclusion. Research staff will also assess the condition of the home environment. Caretakers will receive instructions from research staff on how to collect fecal samples from both the infant and pet dog prior to the scheduled in-home visit. During the in-home study visits, research staff will collect all remaining samples from the infant, pet dog, and home environment.

#### Assessment of adiposity

Infant and pet dog weight change is a primary outcome of interest. Infant length and weight will be measured with portable recumbent length boards (nearest mm) and electronic digital scales (nearest 0.1 kg), respectively. Infants will be nude at the time of weighing and will be measured in triplicate with the average weight taken as the final measurement. The weight of the pet dog will be collected using a Redmon Precision Digital Pet Scale and the body condition score (BCS) will be described using the Nestle Purina Body Condition System for dogs (38).

#### Assessment of temperament

Infant temperament will be measured using the Infant Behavior Questionnaire (39) which is composed of six scales and asks the caretaker to rate the frequency of the infant’s behavior across a variety of situations. The Canine Behavioral Assessment & Research Questionnaire (40) will be used to assess the dog’s behavior across 13 behavioral factors to describe changes in temperament. The caretaker will complete both questionnaires during the in-home study visits and consult study staff if needed.

#### Assessment of diet

An age-appropriate version of the Infant Feeding and Health Questionnaire (41) will be used to collect infant dietary data supplement use, breastfeeding and formula feeding practices, receipt of commercial baby foods, and general health-related questions (41). For pet dogs, dietary information will be collected via a Canine Signalment and Health History Form which will be administered at each study visit. This will include pet owners’ feeding methods.

#### Fecal sample collection

Study staff will mail sample collection kits the week before planned in-home visits with instructions on how to collect the infant and pet dog stool sample the day prior to scheduled in-home visits. Study staff will call and text message caretakers the day prior to the scheduled in-home visit to remind them to collect the infant and pet dog stool samples. Caretakers will swab the infant’s diaper and pet dog stool immediately after defecation using sterile swabs and tubes containing DNeasy buffer. Samples will be stored in a sealed container at −20°C until research staff collect the tubes at scheduled in-home visits. The stool sample will be transferred on dry ice to the laboratory and stored at −80°C until further processing.

#### Saliva, skin, and environmental sample collection

Research staff will collect infant and pet dog saliva samples, infant and pet dog skin samples, and environmental samples during the in-home study visits. Infant and pet dog saliva samples will be collected using SalivaBio collection kits according to the manufacturer protocol (42). Immediately after collection, the swab will be placed in a syringe to express the saliva into a cryovial for storage. The process will be repeated until a 200 ul saliva sample is collected. Research staff will use sterile swabs and tubes to collect samples from the infant’s hands and feet, and pet dog’s paws. One swab each will be used for hands, feet, front paws, and back paws. Research staff will use sterile swabs and tubes to collect samples from environmental surfaces within the home including the floor of the infant and pet dog feeding area, the high-chair surface and seat, the pet dog food and water bowl, and the main indoor play area. One swab will be used for each surface area. All collection swabs will be sealed in a conical tube, transferred on dry ice to the laboratory, and stored at −80°C until further processing.

#### Sample processing and microbial analysis

Microbial genomic DNA will be extracted from fecal, saliva, and environmental samples, as described by the manufacturer (Qiagen Sciences LLC, Germantown, Maryland), using DNeasy PowerSoil Pro DNA isolation kits and a vortex-based beadbeater, the recommended method of the National Institute of Health Human Microbiome Project. Amplification of the 16S rRNA gene sequence will be completed in triplicate PCRs using 96-well plates. The V4 region of the 16S rRNA gene will be amplified by utilizing forward 515F primers and 806R reverse primers containing Illumina adapter sequences. PCR amplification, amplicon cleaning, and quantification will be performed following previously outlined protocols (43). Sequencing will be performed by using the Illumina MiSeq (SN M02149, with the MiSeq Control Software v 2.5.0.5). Pooled sequences will be de-multiplexed and quality filtered using the QIIME2 software package. Sequences will be assigned to operational taxonomic units (OTU) with a 97% similarity threshold using QIIME’s uclust-based open-reference OTU picking protocol against most recent SILVA or GreenGenes reference databases. Sequences that do not match the reference database will be clustered *de novo* including all sequences in the analysis. Core diversity analyses will be performed OTU tables, including alpha and beta diversity and taxonomic summaries as provided by QIIME software. Collaboration with scientists experienced in performing the above-mentioned bioinformatics is necessary for this pilot study.

#### Assessment of covariates

For this pilot study, we will control for gender, age, and diet of the pet dog and human infant in all analyses. Furthermore, we will control for birth mode and breastfeeding status (44) of the human infant in all our analysis as research has demonstrated key differences in the GM of infants based on these factors. In addition to the above-mentioned covariates, for aims 2 and 3, we will also include the following human infant covariates: maternal pre-pregnancy body mass index (45), gestational weight gain (45), and maternal education (46) as previous research have shown their impact on adiposity or rapid infant weight gain among infants. In aims 2 and 3, we will also include covariates related to the most common comorbid conditions in dogs which lead to obesity, hypothyroidism and hyperadrenocorticism (35, 36). There are a number of environmental factors that may impact the health of the infant and pet dog. Several key household factors have been associated with greater risk of obesity among pet dogs including pet owner age, income, and frequency of exercise (25). The number of people residing in the household may impact microbial transfer between infant and pet dog (12). These variables will be assessed during pilot study participant evaluations and controlled for as needed in aims 2 and 3.

Additional factors related to details pertaining to the types of daily interactions that infants have with their pets such as licking/petting/other potential mechanisms of microbiota exchanges outside of feeding times will also be collected during this pilot study. Similarly, information related to animal husbandry practices and habits such as feeding table scraps or allowing a pet to clean the floor under the highchair will be collected through interviews during home visits with the primary adult caregiver. Due to sample size limitations in this pilot study these variables will not include as covariates. The availability and analysis of the resulting data will be utilized as an important tool to inform future research questions and hypothesis for larger follow-on studies.

#### Sample size justification

In microbiome studies, power analyses and sample size estimates can be difficult because the true effect size is unknown. Because of this and that our proposed study is a pilot study, we based our sample size estimate on a recently published protocol paper for an observational study among infants followed through the first 2 years of life which estimated statistical power using simulation yielding a total sample size of 82 (47). This aligns with a sample size calculation using G*Power for ANCOVA with two groups with an *a priori* effect size of 0.47, 80% power, and 8 covariates, yielded a total sample size of 83. Furthermore, Kers and Saccenti recently published statistical power and sample size estimates from simulation of data from the Human Microbiome Project and found to achieve observe differences in microbial features of 25% with 80%, the sample size would range from approximately 40–90 for alpha diversity metrics, and approximately 15–50 for beta diversity metrics (48). Based on these publications, we feel that a total sample size of 82 is sufficient for a pilot study.

#### Statistical plan

All statistical analyses will be completed using Statistical Package for the Social Sciences 25 (SPSS, Inc., an IBM Company, Chicago, Illinois) and QIIME 2020.2 statistical bioinformatics software packages. Data will be expressed as mean ± SD or median (interquartile range) for demographic, anthropometric, temperament scores, and microbiota frequencies based on the normality of the data. Phylogenetic diversity measures will be performed using QIIME 2 longitudinal plug-in to determine alpha (within-sample) diversity via Observed OTUs, Faith’s PD and Shannon’s Index to assess richness and evenness. Median alpha-diversity values will be visually reported using distribution comparison plots (box-and-whisker plots). Beta-diversity (between-sample) metrics to explore community composition will be assessed by principal coordinates analysis (PCoA) plots and visualized using EMPeror (via QIIME 2). Four ß-diversity metrics will be evaluated including Jaccard, Bray-Curtis, Unweighted Unifrac, and Weighted Unifrac. Outliers will be identified using the volatility visualizer to assess the volatility of the dependent variables over the independent variable. Visual identification of outliers that are disproportionately impacting the variance within individuals and groups will be removed. Scatterplots with the metric predictions of each sample on the x-axis and the residual for each sample on the y-axis will be examined to evaluate patterns or non-random distributions. Residuals should be roughly zero-centered and normal across the range of measured values. Non-normal data will be centered log-ratio transformed prior to analysis. To evaluate *Aim 1*, Pearson correlation will be used to examine associations between the GM of the human infant and pet dog with microbial composition and diversity of the skin, saliva, and home environment at each time point with alpha- and beta-diversity metrics for the different microbiome samples analyzed separately. Paired and difference distance comparisons will be evaluated to assess the changes in the gut microbiome between the human infant and pet dog at each time point. Group differences (human infants and pet dogs) will be evaluated by performing linear mixed effects (LME) models at each timepoint (before, during, and after CF) while controlling for diet (human infant and pet dog), gender (human infant and pet dog), age (human infant and pet dog), infant breastfeeding status, and infant birth mode as fixed effects and subject ID as random effect. Alpha- and beta-diversity metrics will be separately analyzed as dependent variables with group assignment as the independent variable. LME models will be fit with all main effect interactions as fixed effects, with insignificant terms removed from the final model for that outcome of interest. All analyses will be performed using the linear-mixed-effects action in the q2-longitudinal plugin for the QIIME 2 microbiome analysis platform. Findings will be considered significant based on a *p* < 0.05 level of each variables Z-score estimate.

To evaluate *Aim 2*, to examine longitudinal changes in microbiomes, LME models will be used to compare changes in pairwise first differences (i.e., magnitude of change) for alpha diversity metrics and pairwise first distances (i.e., rate of change) for beta-diversity metrics while controlling diet (human infant and pet dog), gender (human infant and pet dog), age (human infant and pet dog), infant breastfeeding status, infant birth mode, pre-pregnancy maternal BMI, gestational weight gain, maternal education, canine hypothyroidism and canine hyperadrenocorticism, household income and size, and age and exercise level of main pet caretaker as fixed effects and subject ID as random effect. First difference and distance calculations will be performed at fixed time intervals with the “before CF” timepoint representing the baseline measurement for the human infant and pet dog separately. The pairwise first difference and distance between subjects (human infant and pet dog) will be calculated for a given timepoint and included in the LME models as the independent variable. Subjects that are missing samples for this baseline timepoint will not be included in the analysis. LME models will be used to examine the relationship between the independent variables of interest and the longitudinal response that will include the intercept and slope term as both fixed and random effects. The fixed intercept and slope represent the regression line for the average subject, whereas the random intercept and slope reflect individual departures from the average line for each subject (49). LME models will be fit with all main effect interactions as fixed effects, with insignificant terms removed from the final model for that outcome of interest. All analyses will be performed using the linear-mixed-effects action in the q2-longitudinal plugin for the QIIME 2 microbiome analysis platform. Findings will be considered significant based on a *p* < 0.05 level of each variables Z-score estimate.

To evaluate *Aim 3*, assess whether the changes observed in Aim 2 are correlated with subsequent changes in infant and pet dog adiposity and temperament, we will use the q2-longitudinal’s feature-volatility action. First, we will visually analyze the data by evaluating volatility charts to identify specific timepoints where infant and pet dog temperament or adiposity appears to have a significant impact on microbial diversity. Volatility charts combine the attributes of control charts and spaghetti plots to produce interactive HTML-based visualization where we can select which alpha- or beta-diversity metric is plotted on the y axis, choose the categorical sample metadata column used to group the data. Next, we will use the feature-volatility action to produce an interactive visualization of the longitudinal abundance, importance, and descriptive statistics for all important features. The longitudinal abundance of each feature is plotted using volatility plots (as described above). Important features identified change over time with their abundance predictive of the specific time point when the sample was collected. Finally, we will use LME models (as described in Aim 2) to test whether alpha- and beta-diversity were impacted by changes in adiposity and temperament of the human infant and pet dog including the same covariates as described in Aim 2. For these models, the random effects in the model will include subject ID as the random intercept and timepoint as the slope.

## Discussion

Recently there has been a paradigm shift from the primary focus being the impact animals and the environment have on human health to exploring the unique ecological relationships between humans, animals, and their environments. Both humans and animals harbor distinct microbial communities that colonize a variety of body locations which fulfill a multitude of roles from aiding in digestion to modulation of the immune system (9). There is a wealth of literature that has demonstrated that the gut microbiota are important regulators of health and disease among humans (10, 11), and more recently elucidated, household pets (i.e., a domestic or tamed dog kept for companionship) (1). However, emerging evidence suggests microbiota from other body epithelia (i.e., salivary and skin) also play a major role in mammalian health (1). Despite this, there is a paucity in the literature that has explored the impact of microbial similarities and transfer across the One Health Triad.

In the United States, approximately 12.7% of children ages two to five (50) and one out of three dogs (51) are estimated to suffer from obesity. This is widely considered to be a public health crisis and contributes to increased morbidity and mortality in both humans and animals. Similarly, an estimated one out of every five children in the United States struggle with developmental delays, behavioral, or mental health disorders each year (52). In recent decades, our canine companions have also been more commonly diagnosed with behavioral concerns, some estimates claim that as many as 80% of our indoor canine companions exhibit behavioral problems (53). Greater understanding of these unique ecological relationships will allow researchers to integrate more holistic One Health approaches into future interventions that will inform public health programming. These efforts could potentially lead to improved quality of life for thousands of children and dogs.

Microbial similarities have been demonstrated among humans (13) and pets (14) with their environment, among members of the same household (14, 15), and between pets and their owners (12). Research has shown that the microbiomes of *Canis familiaris* (domestic dogs) are dominated by *Proteobacteria* and *Firmuctes* (39, 54) with the skin containing high abundance of *Actinobacteria*, oral cavity having high abundance of *Bacteroidetes*, and the gut having increased abundance of *Spirochaetes* (54). In comparison, microbial composition vary across infant body sites with the GM primarily colonized by *Firmicutes* and *Bacteroidetes*, the skin containing *Bacilli*, *Clostridia*, and *Actinobacteria* (55) but also environmentally derived microbes (56) (*Massila*, *Streptococcus alactolyticus*, and *Methylobacterium mesophilicum*), and the oral microbiota dominated by *Lactobacillus* and *Staphylococcus* (57). Interestingly, research has shown that more confined living environments have a greater effect on an inhabitants’ microbiome (13, 14). Pets have been shown to promote microbial exchange between cohabiting humans (11) which may be due to the shared environment (20) and greater physical contact (21). Cohabitation affects microbial composition of both pets and humans across a variety of body epithelia; however, the unique microbial exchange across the One Health Triad impact on health is only beginning to be elucidated.

Infancy marks an important period of growth and development as introduction of solid foods is associated with shifts in the dominant bacterial phyla present in the infant gut (16, 17) resulting in increased microbial diversity and a more stable GM (19). Infants have a unique relationship with their pets due to greater environmental exploration and similar means of mobility leading to more opportunities for microbial exchange. Perturbations in microbial composition of various body sites are associated with a wide range of human health issues including adiposity (23, 24), behavioral problems (8), and temperament (11). Research has found that pet ownership enriched the abundance of *Oscillospira* and *Ruminococcus* among infants, bacterial genera negatively associated with childhood obesity and atopy, respectively (22). Similarly, microbial dysbiosis among pet dogs is associated with aggressive and phobic behaviors (25, 26), and overweight status (20). However, whether there are similar health benefits to the animal and for other inflammatory diseases (i.e., adiposity) has not been extensively researched. Therefore, it is important that we examine the impact ecological relationships have on microbial colonization and how transfer across the One Health Triad impacts the health of both the animal and infant.

One Health approaches such as this proposed pilot study have the potential to reveal new information about complex public health problems and hopefully inform cutting edge interventions for issues such as obesity and behavioral health which continue to plague modern society. The intention of this pilot study is to continue to shed light through a One Health lens into the landscape of the ever-growing field of microbiome research and inform future holistic public health initiatives and interventions.

## Study limitations

The small sample size of this pilot study results in several limitations. A primary restriction is the number of covariates we will be able to include in our proposed analyses methods. Our hope is that findings from this pilot study will inform future research including the impact that parents and other members of the household may have on microbiota exchange between infants and pet dogs. Additionally, the impact of differential exposures prior to the complementary feeding period such as licking, petting, and other interactions that can occur during initial tummy time when infants are often placed on the floor of homes can be potential confounders or biases in this study. This life stage is not the primary focus of this study and therefore, not included in our aims or methods and presents an opportunity for future research. Care givers’ and pet owners’ feeding methods and dietary choices will not be included in this study. Infant complementary feeding method variation such as purees vs. infant led weaning and variation in animal husbandry behaviors such as feeding raw pet foods and or table scraps or allowing a pet to clean up around the highchair after an infant’s meals are additional potential confounding factors that should be considered in future studies. Our hope is that findings from this pilot study will inform future research including the possible impact that these other potentially confounding variables may have on microbiota exchange between infants and pet dogs.

## Ethics statement

The authors intend to register this pilot study with ClinicalTrials.gov prior to undertaking clinical research. Furthermore, the Midwestern University IRB and IACUC have determined that this pilot study protocol does not currently meet the definition of human subjects or animal research. Prior to undertaking the pilot study outlined within this protocol, a full IRB and IACUC review will be completed.

## Author contributions

KV contributed the initial idea, inspiration, and outline for this protocol and researched and wrote the portions of the pilot study protocol relevant to human subjects as well as sample and data analysis. MZ served as a subject matter expert for the environmental and animal aspects of the One Health triad and KV for microbiome and human health and researched and wrote the portions of the pilot study protocol relevant to animal and environmental sampling. KV and MZ partnered and were equal contributors in writing the final manuscript. All authors contributed to the article and approved the submitted version.

## Conflict of interest

The authors declare that the research was conducted in the absence of any commercial or financial relationships that could be construed as a potential conflict of interest.

## Publisher’s note

All claims expressed in this article are solely those of the authors and do not necessarily represent those of their affiliated organizations, or those of the publisher, the editors and the reviewers. Any product that may be evaluated in this article, or claim that may be made by its manufacturer, is not guaranteed or endorsed by the publisher.
